# Flow-Based Coronary Artery Bypass Graft Patency Metrics: Uncertainty Quantification Simulations to Guide Development

**DOI:** 10.1007/s13239-024-00765-8

**Published:** 2025-01-03

**Authors:** Sita Drost, Cornelis J. Drost

**Affiliations:** 1 Transonic Europe B.V., Business Park Stein 205, Elsloo, 6181 MB The Netherlands; 2 Transonic Systems Inc., 34 Dutch Mill Road, Ithaca, New York 14850 USA

**Keywords:** CABG, Computational fluid dynamics, Hemodynamics, Sensitivity analysis, TTFM, Uncertainty quantification

## Abstract

**Purpose:**

Over time, transit time flow measurement (TTFM) has proven itself as a simple and effective tool for intra-operative evaluation of coronary artery bypass grafts (CABGs). However, metrics used to screen for possible technical error show considerable spread, preventing the definition of sharp cut-off values to distinguish between patent, questionable, and failed grafts. The simulation study presented in this paper aims to quantify this uncertainty for commonly used patency metrics, and to identify the most important physiological parameters influencing it.

**Methods:**

Uncertainty quantification was performed on a realistic multiscale numerical model of the coronary circulation, guided by Morris screening sensitivity analysis of a simpler, lumped-parameter model. Simulation results were qualitatively verified against results of a recent clinical study.

**Results:**

Correspondence with clinical study data is reasonable, especially considering that the model was not fitted in any way. Stenosis severity was confirmed to be an influential parameter. However, also cardiac period and graft diameter were observed to be important, particularly for mean flow rate and pulsatility index.

**Conclusion:**

Metrics quantifying the flow waveform’s diastolic dominance show the highest sensitivity to graft stenosis, and seem to be least affected by autoregulation. Among these, the novel diastolic resistance index shows the strongest sensitivity to stenosis severity.

**Significance:**

The approach used in this study is expected to benefit the development of improved patency metrics, by allowing medical engineers to include sensitivity and uncertainty in assessing, in-silico, the potential of novel metrics, thus enabling them to provide better guidance in the design of clinical studies.

## Introduction

Coronary artery bypass grafting (CABG) is a surgical procedure with a high rate of success. Still, a small percentage of grafts is reported to fail in the period up to 12 months post-surgery [1–3]. Reasons for graft failure are multitudinous, but among them is technical error. Therefore, to detect and correct technical error before chest closure, and thus reduce the risk of subsequent graft failure, intraoperative graft patency assessment is generally considered a valuable addition to the CABG surgery procedure [1, 4].

The gold standard for detecting flaws in a graft and its anastomosis is coronary angiography (CAG) [5, 6]. Angiography provides a precise assessment of vascular constriction; a qualitative assessment of the graft’s capacity to carry flow. The measurement involves relatively costly surgical equipment, requiring a trained operator and added surgical time, and is therefore not widely used for intraoperative patency assessment [1].

As a faster, simpler, and more affordable alternative, ultrasound transit time flow measurement (TTFM), as originally developed by Transonic Systems Inc. [7], is increasingly used for intraoperative CABG patency assessment [1–4]. By placing a hand-held flow probe around a newly created graft and holding it still for several seconds, the surgeon can accurately measure the time-varying flow rate through the graft. Deviations in the measured flow waveform can alert the surgeon of possible technical imperfections before the patient’s chest is closed. Presently in Japan, all CABG procedures include some form of flow-based patency assessment [8], while in the United States this percentage lies around 20% [4], the majority of which concerns off-pump CABG surgery. Also, the European 2018 ESC/EACTS Guidelines on Myocardial Revascularization [9] recommend TTFM to confirm graft patency.

The measured waveform is summarized in a number of patency metrics (see Sect. 2.1 for details), which were established over the years, based on clinical experience. Ideally, a (combination of) metric(s) should identify correctable technical errors in the newly constructed graft, independent of surgical confounders and patient parameters. However, in practice flow-based patency assessment is complicated by the many factors influencing the CABG flow waveform, such as target coronary, graft type (e.g. arterial or venous, single or sequential), competitive flow (resulting from incomplete occlusion of native coronary), autoregulation, quality of coronary microvasculature (resistance, compliance), but also patient parameters such as disease history, heart rate, cardiac index, blood pressure, and BMI [10]. As a result, the reliability of most flow-based patency metrics is high for the identification of severe stenoses, but decreases in case of subcritical stenoses [11].

Accounting for these factors in the metrics displayed on a TTFM flow monitor, or developing a novel metric, would require years of carefully controlled clinical studies. Development of novel metrics is ongoing [11–13], and generally is an empirical process, based on experience and clinical studies. To better guide such developments, a basic understanding is needed of the mechanisms determining the graft flow waveform and the factors influencing it. At the same time, such basic understanding is also expected to facilitate the user’s interpretation of a measured waveform on existing flow monitors. Therefore, as a complement to knowledge gained from clinical studies, we use computational fluid dynamics models to improve our understanding of the graft flow waveform.

The aim of our present study was to quantify uncertainty in the most commonly used patency metrics, and thus to assess their performance. To this end, we implemented a validated multi-scale numerical model of the coronary circulation [14–16], customized to include a CABG graft. To select parameters for uncertainty quantification using this model, Morris screening sensitivity analysis was performed using a simpler lumped-parameter model. Results were qualitatively verified against data from a recent clinical study by Takahashi et al. [11]. By providing an indication of the epistemic uncertainty associated with a certain metric, and of the key physiological parameters influencing this uncertainty, our approach should facilitate the design of effective clinical studies, and thus accelerate and focus the ongoing development of improved CABG patency metrics.

Sensitivity analysis and uncertainty quantification on a similar model, but applied to the pulmonary circulation, were reported by Colebank et al. [17], while Ge et al. [18] did a more limited, one-at-a-time (OAT) sensitivity analysis on a similar model of the coronary circulation. To the authors’ knowledge, this study is the first to specifically target flow-based CABG patency assessment.

## Methods

**Fig. 1:**
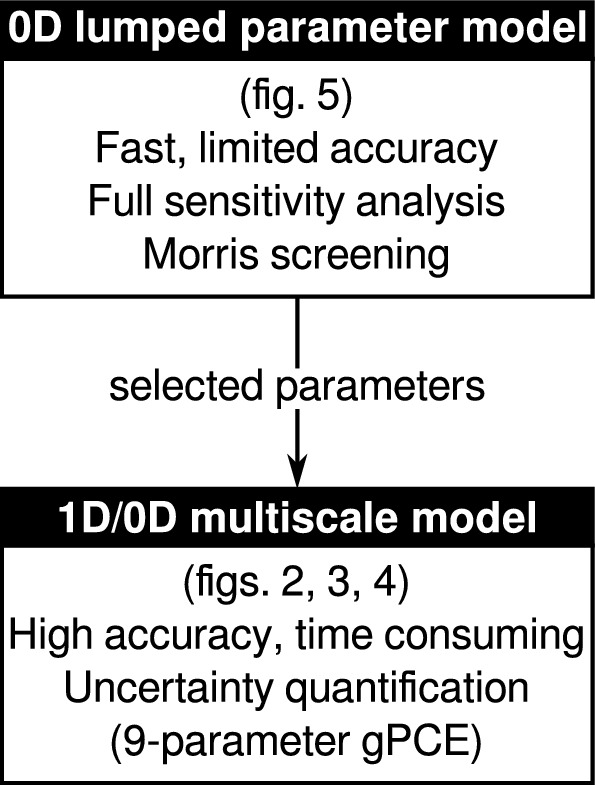
Flow chart representing the methods used in this study: first, Morris screening sensitivity analysis was performed, using a low-fidelity lumped parameter model. The parameters indicated as most influential by this analysis were used for generalized polynomial chaos expansion (gPCE) uncertainty quantification, using a realistic multiscale model.

**Fig. 2:**
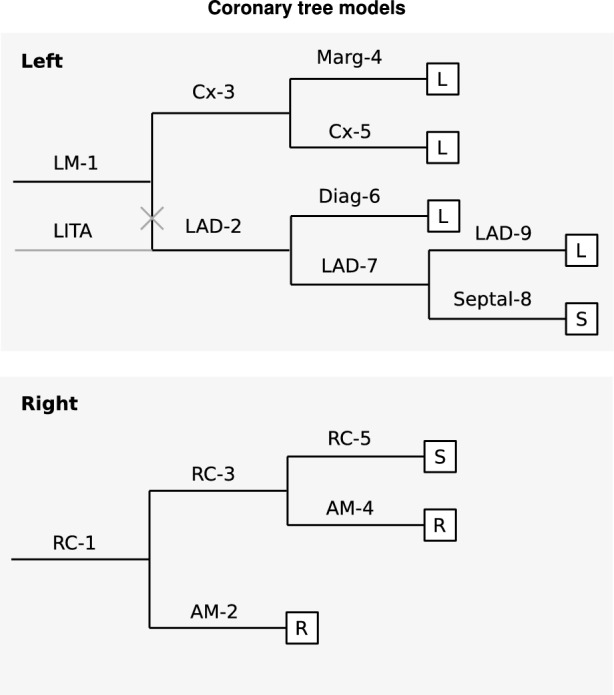
**1D/0D multiscale model:** Diagrams detailing structure of left (top) and right (bottom) coronary tree models [14]. Labels correspond with segment names in Supplementary Material, boxes with L, R, S, represent instances of the lumped-parameter intramyocardial circulation model (Fig. 4) in the left and right ventricular wall and septum, respectively. The grey segment indicates a LITA graft, connected to the LAD (complete occlusion of LAD proximal to anastomosis is assumed in simulations with graft).

**Fig. 3:**
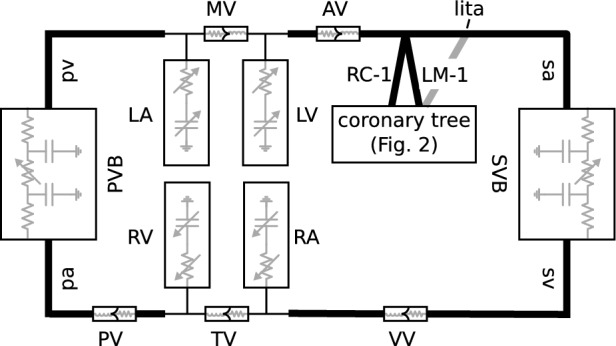
**1D/0D multiscale model:** Closed-loop cardiovascular model with coronary tree (adapted from Mynard et al. [16]). Boxes represent lumped-parameter models of microvascular beds, heart chambers, and valves, thick lines represent 1D segments, thin lines represent connections to lumped parameter models. LA/RA/LV/RV: left/right atrium/ventricle; MV: mitral valve, AV: aortic valve; TV: tricuspid valve; PV: pulmonary valve; VV: venous valve; PVB/SVB: pulmonary/systemic microvascular bed; sa: systemic artery; sv: systemic vein; pa: pulmonary artery; pv: pulmonary vein; LM-1: left main coronary artery; RC-1: right coronary artery, lita: left interior thoracic artery graft.

**Fig. 4:**
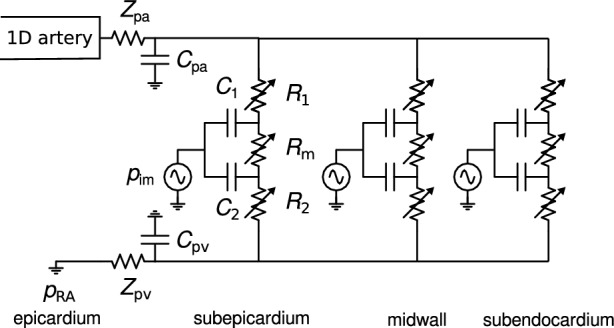
**1D/0D multiscale model:** Lumped-parameter model of intramyocardial circulation (adapted from Mynard et al. [16]). Intramyocardial pressure *p*$_\text {im}$ is generated by ventricular contraction, its influence is set to increase linearly with depth in the myocardial wall.

**Fig. 5:**
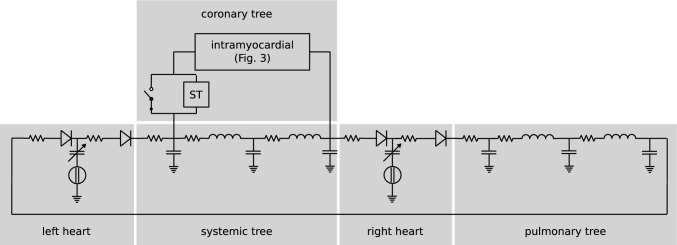
**0D lumped parameter model:** Lumped-parameter cardiovascular circulation model of Avanzolini et al. [28], with one instance of the intramyocardial circulation model of Mynard et al. [16] connected to it to represent coronary circulation. ST: stenosis model (3) [23], enabled by opening switch. See Appendix C for details.

To study the effects of stenosis in a graft, we use a realistic multi-scale numerical model of the coronary circulation (Figs. 2 and 4), embedded in a closed-loop cardiovascular model (Fig. 3), both developed by Mynard et al. [14, 16] and validated in-vivo [15]. Because, depending on stenosis severity and other parameter values, simulations with this model take between 5 and 100 h, performing a global sensitivity analysis is not feasible with this model. Instead, generalized polynomial chaos expansion (gPCE) uncertainty quantification was performed, with a limited number of parameters. To guide parameter selection, Morris screening sensitivity analysis was performed with a simpler – and thus, faster – lumped-parameter model (Fig. 5). The overall set-up our study is explained schematically in Fig. 1.

### Patency Metrics

In this study, the four most commonly used TTFM CABG patency metrics were included:

1a $$\begin{aligned}&\text {Mean\, flow\, rate:}&\quad &Q_\text {mean} = \frac{1}{T}\int _0^T Q(t) \textrm{d}t, \end{aligned}$$

1b $$\begin{aligned}&\text {Pulsatility\, index:}&\quad &\text {PI} = \frac{Q_\text {max} - Q_\text {min}}{Q_\text {mean}}, \end{aligned}$$

1c $$\begin{aligned}&\text {Diastolic/systolic\, ratio:}&\quad &\text {D/S-ratio} = \frac{|V_\text {dia}|}{|V_\text {sys}|}, \end{aligned}$$

1d $$\begin{aligned}&\text {Diastolic\, filling\, percentage:}&\quad &\text {DF\%} = \frac{|V_\text {dia}|}{|V_\text {tot}|}. \end{aligned}$$

Here, *Q* is flow rate, *T* is cardiac period, *V* is delivered blood volume (based on absolute flow rate), and subscripts max, min, tot, sys, and dia indicate maximum, minimum, total, systolic, and diastolic, respectively. DF% and D/S-ratio are directly related, as $\text {DF\%} = 100 \times \text {D/S-ratio}/(1 + \text {D/S-ratio})$. Therefore, DF% was not included in the figures presented here.

Additionally, diastolic resistance index (DRI) was included:

2 $$\begin{aligned}&\text {Diastolic\, resistance\, index:}&\quad &\text {DRI} = \frac{\overline{p}_\text {dia}/|\overline{Q}_\text {dia}|}{\overline{p}_\text {sys}/|\overline{Q}_\text {sys}|}, \end{aligned}$$

where *p* is (central) pressure. This novel metric, developed by Transonic Systems Inc. (Ithaca, NY), was recently evaluated in a first clinical study by Takahashi et al. [11]. It incorporates both flow rate and pressure, and is related to D/S-ratio as:

$$\begin{aligned} \text {DRI} = \frac{\overline{p}_\text {dia}}{\overline{p}_\text {sys}}\frac{T_\text {dia}}{T_\text {sys}}\frac{1}{\text {D/S-ratio}}. \end{aligned}$$

It should be noted that, because DF% and D/S-ratio are computed using the absolute value of the flow rate, the same is done for DRI.

As explained in more detail in Appendix A, these patency metrics quantify different aspects of how the flow waveform changes in the presence of stenosis: $Q_\text {mean}$ quantifies the overall decrease in flow rate, while D/S-ratio, DF% and DRI capture the decrease in diastolic dominance. Finally, PI quantifies the combined effect of increasing capacitive flow proximal to the stenosis, and decreasing mean flow rate.

### 1D/0D Multiscale Model

The high-accuracy multiscale model that was used for gPCE uncertainty quantification consists of a model of the coronary circulation (Fig. 2), embedded in a closed-loop cardiovascular model (Fig. 3). Both the coronary tree model and the closed-loop cardiovascular model are made up of 1D segments representing the coronary arteries, and main systemic and pulmonary arteries and veins. The coronary artery segments are terminated with 0D lumped parameter intramyocardial perfusion models, and also the pulmonary and systemic vascular beds, and the heart were represented by 0D lumped parameter models.

In the lumped-parameter intramyocardial perfusion model (Fig. 4), the resistances in the sub-epicardial, mid-wall, and sub-endocardial layers depend on vascular volume. Incorporating volume-dependence of the compliances in these layers, as outlined by Algranati et al. [19], did not significantly alter the results, while it did increase the number of uncertain parameters in the model. Therefore, the compliances were assumed to be fixed in our study.

Following Suga et al. [20], maximum elastance in the heart model was set to be independent of cardiac period, while for diastole duration as a function of cardiac period the relation determined by Salvi et al. [21] was implemented.

To this model a left internal thoracic artery (LITA) graft was added (indicated in grey in Fig. 2). Length and diameter of the LITA graft were based on data from the anatomically detailed arterial network (ADAN) model [22], resulting in a 20 cm long graft, with a radius tapering linearly from 0.142 cm to 0.0837 cm. This graft was anastomosed onto the left anterior descending (LAD) coronary artery, 0.3 cm distal to its origin from the left main (LM) coronary artery. Proximal to the anastomosis, the LAD was fully occluded. The origin of the LITA was placed on the systemic artery segment, 8 cm from the coronary ostia, so that the delay and distortion of its pressure waveform matched the ADAN data.

To the LITA graft, a stenosis was added at 1 cm from its distal end (model structure did not allow for stenosis to coincide with the anastomosis). Graft flow rate and pressure were monitored at two sites: 1 cm and 5 cm proximal of this stenosis. The former position corresponds with the flow probe position that is recommended by flow probe manufacturers, whereas the latter seems to better agree with clinical practice (based on informal discussions).

Stenosis was modeled following Young and Tsai [23]:

3 $$\begin{aligned} \Delta p_\text {s} = \frac{4 K_\text {v} \mu }{\pi D_0^3} Q_\text {s} + \frac{\rho K_\text {t}}{2 A_0^2}\left( \frac{A_0}{A_\text {s}} - 1\right) ^2 Q_\text {s}^2 + \frac{\rho K_\text {u} l_\text {s}}{A_0} \frac{\textrm{d}Q_\text {s}}{\textrm{d}t}, \end{aligned}$$

where $\Delta p_\text {s}$ is the pressure drop over the stenosis, $D_0$ and $A_0$ are the un-stenosed vessel diameter and cross-sectional area, respectively, $A_\text {s}$ and $l_\text {s}$ are the cross-sectional area and effective length of the stenosis, respectively, and $K_\text {v}$, $K_\text {t}$ and $K_\text {u}$ are empirical constants related to viscous, turbulent, and inertial effects, respectively. The values of $K_\text {t} = 1.52$ and $K_\text {u} = 1.2$ are independent of stenosis shape, whereas $K_\text {v}$ depends on stenosis geometry as

$$\begin{aligned} K_\text {v} = 32 \frac{0.83 l_\text {s} + 1.64 D_\text {s}}{D_0}\left( \frac{A_0}{A_\text {s}}\right) ^2, \end{aligned}$$

with $D_\text {s}$ the diameter of the vena contracta. To study the effects of stenosis on graft flow, preliminary simulations were carried out, with 0, 50, 75, and 90% graft diameter reduction (see Appendix A).

Even though arterial pressure is known to be influenced by manipulation of the heart during off-pump CABG surgery [24–26], insufficient quantitative data were available to reliably include these changes in our simulations.

Finally, to limit model complexity, chamber interaction, as implemented by Mynard et al. [14, 16], and autoregulation, as implemented by Ge et al. [18], were not included in most simulations (however, see Sect. 2.6). Full model details and parameter values can be found in Appendix B.

The model was implemented in Python (https://www.python.org), using a finite difference flux-limited MacCormack scheme [27] to discretize the 1D flow equations. After checking for grid independence, a grid size of 0.1 cm was selected, with a time step of $2\times 10^{-5}$ s for simulations without stenosis, and progressively smaller for increasing stenosis severity. To simulate 10 cardiac periods, this resulted in simulation duration ranging from around 5 to 100 h (Dell Optiplex-7070 with Intel Core i7 processor).

### 0D Lumped Parameter Model

Because, based on earlier, exploratory simulations, most of the changes in the flow waveform as a result of stenosis were observed to originate from the lumped parameter stenosis model and the coronary microvasculature, a simplified model was created by connecting a single instance of the lumped parameter model of the intramyocardial vessels (Fig. 4) to the closed-loop lumped parameter cardiovascular circulation model of Avanzolini et al. [28] (Fig. 5). In a lumped parameter model, the geometric and mechanical properties of blood vessels are represented by lumped resistance, compliance, and (in larger vessels) inductance components. This way, a model comparable to that of Mantero et al. [29] was obtained, with which all essential features of the coronary flow waveform could be reproduced (see e.g. Fig. 13 in appendix C), including changes in myocardial perfusion as a function of diastolic time fraction [30] and perfusion pressure [19]. This model, with 39 parameters (including stenosis percentage), was implemented in OpenModelica (https://openmodelica.org/), and then exported as a functional mock-up unit (FMU), to be called from Python. With around 0.5 s to simulate 10 cardiac periods, this model allowed for global sensitivity analysis using Morris screening [31].

### Sensitivity Analysis

For the Morris screening sensitivity analysis, the Python package SALib [32, 33] was used, in which the efficient sampling strategy proposed by Ruano et al. [34] is implemented. For two parameters – stenosis percentage and subendocardial-subepicardial volume ratio [35] – a uniform distribution was assumed. For all other parameters, a normal distribution was assumed, truncated to mean $\pm 2\times$standard deviation, with mean and standard deviation values guided by literature, such as the work of Charlton et al. [36]. If no standard deviation could be found in literature, a value of 25% of the mean of that parameter was assumed. For Morris screening, the parameter value distributions were sampled at $p = 4$ levels, and $r = 50$ trajectories were computed, resulting in a total of 2000 simulations (*r* was determined by increasing it in steps of 10, until the resulting 3 most influential parameters stopped changing). The patency metrics presented in the previous subsection, $Q_\text {mean}$, PI, D/S-ratio, and DRI, were used as response variables. The influence of a parameter, quantified by the sensitivity measures $\mu ^*$ (mean of absolute elementary effects) and $\sigma$ (standard deviation of elementary effects), was summarized by $\sqrt{\mu ^{*2} + \sigma ^2}$.

To ensure that the variables with uniform distribution did not have more influence just because the uniform distribution has higher entropy than the normal distribution, the sensitivity analysis was repeated with uniform distribution for all variables (with values in same range as in the corresponding truncated normal distribution). The influence of using all uniform distributions was found to be limited, and did not change the ranking of the most influential parameters.

### Uncertainty Quantification

Apart from stenosis severity, the six most influential parameters resulting from Morris screening were selected for uncertainty quantification of the multiscale model. In addition to these parameters, the cross-sectional area and pulse wave speed of the systemic artery and graft were included. These were not part of the simplified lumped parameter model, but are known to influence the graft flow waveform and systemic pressure, respectively [15]. To limit the number of required simulations, only a model with LITA-LAD graft was analyzed, as this is the most commonly applied graft.

For the resulting parameters, a generalized polynomial chaos expansion (gPCE) was computed using Dakota [37]. Normal distributions were assumed for all parameter values but percentage stenosis, for which a uniform distribution was assumed.^1^ To prevent unrealistic values, normally distributed parameters were constrained to [mean value $\pm 1.96\times$standard deviation] (i.e. 95% of probability density function). Using a maximum polynomial order of 2 (based on exploratory, coarse grid UQ with maximum polynomial order 4), and least-squares regression with a collocation factor of 2, this resulted in 110 simulations. As a part of the analysis, Dakota tested the quality of the gPCE using *k*-fold cross-validation, with 10 folds. To quantify the sensitivity of the flow-based metrics to each parameter, Sobol indices were computed directly from the resulting best-fitting gPCE [37].

Because parameters were assumed to vary independently, not all resulting flow and pressure waveforms were physiologically realistic. Using healthy subject values as a reference, as done by Charlton et al. [36], seemed too restrictive, as such values can hardly be expected to be representative of patients undergoing CABG surgery. Also, the availability of detailed physiological data of CABG surgery patients is insufficient to filter out unrealistic results. Therefore, all Morris screening results were retained, while from the gPCE results only the obviously erroneous results, with maximum pressure during diastole, were removed. This resulted in the removal of 6 out of 110 simulations (5%).

### Autoregulation

In a normally functioning heart, autoregulation dilates or contracts the vessels in the myocardial wall, depending on flow demand [30]. As a result, blood flow to the myocardium, and subendocardial-to-subepicardial flow ratio, are not significantly affected in the presence of a coronary stenosis up to about 68% (90% area reduction, see e.g. Buss et al. [38]). Therefore, we expect the patency metrics to be affected by autoregulation.

As mentioned earlier, autoregulation was not included in the sensitivity analysis and uncertainty quantification simulations, to restrict computational cost. To still get an impression of the effects of autoregulation, four simulations with a stenosed LITA graft were run separately, in which intramyocardial resistance was tuned iteratively to maintain intramyocardial flow rate and endo-epi ratio at the un-stenosed level. These simulations were run with 20, 40, 50, and 60% stenosis, respectively.

### Verification with Clinical Data

Dr. Takahashi of Nippon Medical School, Tokyo, Japan, kindly shared the raw data from his recent clinical study [11], which consisted of graft flow rate, radial artery pressure, and ECG waveforms, measured intraoperatively in 123 grafts, in 41 patients undergoing off-pump CABG surgery. From these data, $Q_\text {mean}$, PI, D/S-ratio, DF%, and DRI were determined. Additionally, in all grafts percentage stenosis was measured postoperatively, using coronary computed tomography (CCT, for correspondence between measured stenosis grades, patency classes, and percentage diameter reduction, see Appendix, Table 8). The ECG signal was used for systole and diastole detection. Central systolic and diastolic pressure were estimated from the radial artery pressure waveform using the *N*-point moving average method proposed by Xiao et al. [39]. For measurement protocol, see Appendix, Sect. D.1.

For each patency metric, the median and interquartile range (IQR) for three different patency classes were compared between the clinical and simulated (1D/0D model) results. Because of the lack of detailed physiological data in CABG surgery patients, the 1D/0D model results were not expected to show quantitative agreement with the clinical results. Therefore, although the nonparametric Wilcoxon’s rank sum test was used to test for the significance of any observed differences, comparison was mostly qualitative, focusing more on trends than on exact agreement.

## Results

### Morris Screening and Uncertainty Quantification

Based on the results of the Morris screening analysis (see Appendix E), systemic vascular bed reference resistance $R_\text {0,SVB}$ (i.e. peripheral resistance), intramyocardial compliance arterial side $C_1$, intramyocardial reference volume arterial side $V_{0,1}$, and cardiac period *T* were selected for uncertainty quantification of the multi-scale model. Two additional parameters, maximum and minimum left ventricular elastance, were not selected for uncertainty quantification, as they were observed to mainly influence systemic pressure. Because systemic pressure is generally more influenced by cross-sectional area $A_{0,\text {sa}}$ and pulse wave velocity $c_{0,\text {sa}}$ of the systemic arteries [16] (not included in the lumped-parameter model), these parameters were selected instead. Finally, also the cross-sectional area and pulse wave velocity of the LITA graft, $A_\text {0,LITA}$ and $c_\text {0,LITA}$, respectively, were included.

**Fig. 6:**
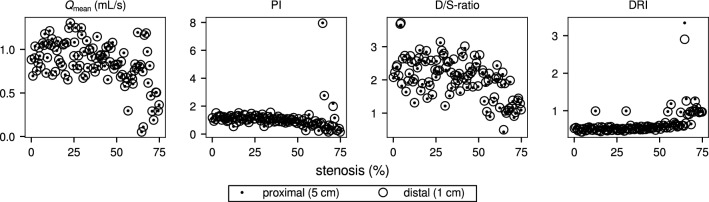
Raw results of uncertainty quantification simulations, plotted as a function of percentage stenosis for each patency metric (black dots: 1 cm from stenosis at distal end of graft, open circles: 5 cm from stenosis at distal end of graft); for PI, D/S-ratio and DRI, differences between proximal and distal measurements are small but statistically significant.

Scatter plots of the resulting patency metrics’ values vs. percentage stenosis, as monitored at 5 cm and 1 cm from the stenosis, are shown in Fig. 6. The observation by Jelenc et al. [40], that diastolic dominance is consistently lower when measured on the proximal side of the graft, is confirmed by our simulation results: D/S-ratio is on average 1.4% lower at 5 cm than at 1 cm from the stenosis (Wilcoxon signed rank test: significant difference, $p < 0.001$). Significant differences were similarly seen for DF% (0.4% lower), DRI (1.6% higher), and PI (2.7% lower).

Based on 10-fold cross-validation, linear expansion (i.e. order 1) was found to give the smallest error for all metrics. That is, even though the number of simulations allowed for second-order expansion, linear expansion was used for the gPCE. The Sobol indices computed from the gPCE, monitored at 5 cm from the stenosis, are listed in Table 1, with the most influential parameters printed in bold (see Appendix, Table 11 for results at 1 cm). Because linear expansion was used in all cases, the main and total Sobol indices are equal, so that only one value is given in the table.

**Table 1:**
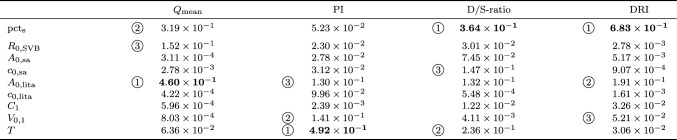
Sobol indices from 9-parameter gPCE (5 cm from stenosis at distal end of graft); circled numbers indicate ranking

pct$_\text {s}$: percentage stenosis; $R_\text {0,SVB}$: reference resistance systemic vascular bed; $A_\text {0,sa}$: unstressed cross section systemic artery; $c_\text {0,sa}$: unstressed wave speed systemic artery; $A_\text {0,lita}$: unstressed cross section LITA graft; $c_\text {0,lita}$: unstressed wave speed LITA graft; $C_1$: intramyocardial compliance (arterial side); $V_{0,1}$: reference intramyocardial volume (arterial side); *T*: cardiac period

### Autoregulation

**Fig. 7:**
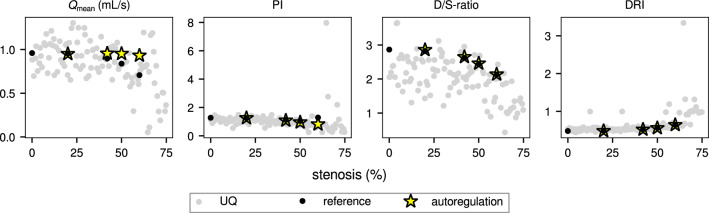
Autoregulation results (yellow stars), and corresponding reference results (i.e. same parameter values, without autoregulation; black dots), plotted as a function of percentage stenosis (uncertainty quantification results are shown as grey dots in background; all results were monitored at 5 cm from stenosis at distal end of graft). As intended, autoregulation prevents $Q_\text {mean}$ from decreasing with increasing stenosis; the other metrics are only slightly (PI), or not noticeably (D/S-ratio, DRI) affected by autoregulation.

The results of the four autoregulation simulations, together with their reference results (i.e. same parameters, without autoregulation) are superimposed onto the raw uncertainty quantification results in Fig. 7. As intended, mean flow rate remains practically unchanged, while PI decreases slightly with stenosis severity, and both D/S-ratio and DRI are not noticeably affected by autoregulation.

### Verification Against Clinical Study Results

**Fig. 8:**
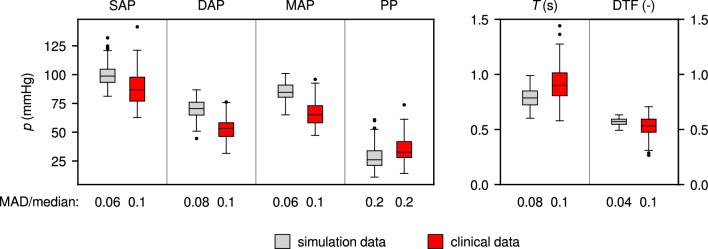
Hemodynamic and cardiac parameters in 1D/0D simulation results (grey) and NMS clinical study results (red) [11]. SAP: systolic arterial pressure, DAP: diastolic arterial pressure, MAP: mean arterial pressure, PP: pulse pressure (mmHg, all in ascending aorta), *T*: cardiac period (s), DTF: diastolic time fraction (-); MAD/median quantifies parameter variability. Clinical study values of SAP, DAP and MAP are significantly lower than simulation results, PP and T are significantly higher.

**Fig. 9:**
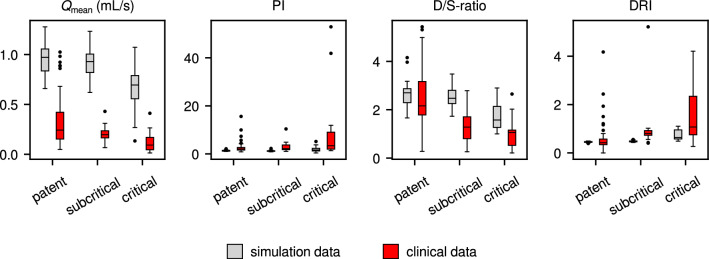
Bar plots of flow-based metrics’ values in 1D/0D simulation results (grey) and NMS clinical study results (red), grouped per graft patency class (monitored at 5 cm from stenosis at distal end of graft). All metrics except PI show the same trend; except for $Q_\text {mean}$, quantitative agreement is reasonable.

Box plots of hemodynamic parameters (systolic, diastolic, and mean arterial pressure in ascending aorta, with corresponding pulse pressure), and cardiac parameters (cardiac period, diastolic time fraction) are shown in Fig. 8. To quantify the variability of these parameters, the value of the median absolute deviation (MAD) relative to the median [41] is given under each box. The corresponding flow-based metrics’ values are shown in Fig. 9. The clinical study values of SAP, DAP and MAP are significantly lower than those in the simulation results (Wilcoxon’s rank-sum test), while PP and *T* are significantly higher. The variability of all hemodynamic and cardiac parameters is slightly higher in the clinical study results, or even much higher, as in the case of diastolic time fraction.

The 1D/0D simulation results for $Q_\text {mean}$, D/S-ratio, and DRI show the same trend as the clinical results, but for PI, the trend is in opposite direction, and only the outliers suggest an increase with increasing stenosis severity. Furthermore, the clinical study values of $Q_\text {mean}$ are much lower than those in the simulations. All metrics’ values show a larger variability in the clinical study results.

## Discussion

**Fig. 10:**
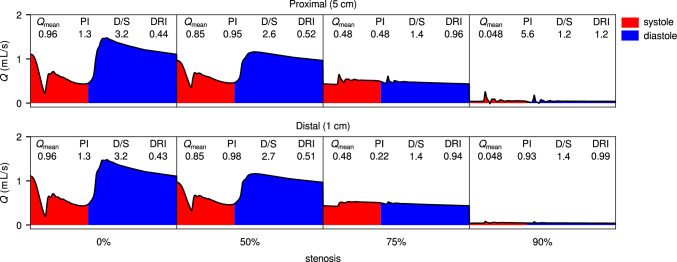
LITA flow waveforms (red and blue indicate systole and diastole, respectively) with corresponding patency metrics, for increasing stenosis percentage, measured proximally and distally (i.e. 5 cm and 1 cm from stenosis at distal end of graft, respectively). With increasing stenosis percentage, diastolic dominance decreases, and difference between proximal and distal waveforms becomes more pronounced. D/S-ratio, DRI, and especially PI are affected by this difference.

**Fig. 13:**
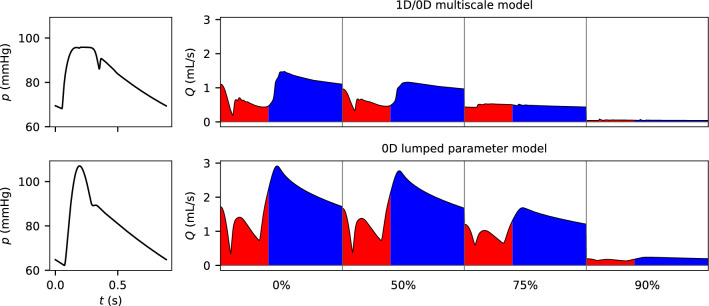
Top: results obtained with 1D/0D multiscale model, using default parameter values (see appendix B). Top left: ascending aorta pressure, top right: flow waveforms on distal side of LITA graft, at different degrees of stenosis. Bottom: results obtained with 0D lumped parameter model, using default parameter values, except for $T_\text {c}$, which was set to 0.89 s to match the 1D/0D multiscale model. Bottom left: ascending aorta pressure, bottom right: coronary flow waveforms at different degrees of stenosis (measured in $Z_\text {pa}$, see Fig. 4). Waveforms and stenosis dependence show satisfactory agreement in terms of order of magnitude and trends.

Uncertainty quantification of the multiscale model, using generalized polynomial chaos expansion (gPCE), showed that for D/S-ratio and DRI, percentage stenosis is the most influential parameter, while for $Q_\text {mean}$ and PI, $A_\text {0,lita}$ and *T* are most important, respectively. Considering the inverse square relation between graft resistance and $A_\text {0,lita}$, the strong influence of the latter on $Q_\text {mean}$ is understandable. The low sensitivity of PI to pct$_\text {s}$ found here might be explained by the fact that stenosis severity was limited to a maximum of 75% in our simulations, while PI only seems to start consistently increasing at pct$_\text {s}$ even higher than that (e.g. Fig. 10). For a metric to reliably detect stenosis, a high Sobol index for pct$_\text {s}$ is needed, preferably combined with low Sobol indices for other parameters of influence. During CABG surgery, $A_\text {0,lita}$ and *T* are routinely monitored so that it should be possible to account for their influence.

In the presence of autoregulation, intramyocardial arterioles will dilate to compensate for a reduction in perfusion pressure. As a result, stenoses with less than 90% area reduction (i.e. 68% diameter reduction) hardly affect mean flow rate and endo/epi ratio [38]. Therefore, in clinical practice, $Q_\text {mean}$ is generally observed to be useful for the detection of severe stenosis only. Our preliminary autoregulation simulations showed that also the utility of PI decreases. The diastolic dominance-based metrics D/S-ratio, DF% and DRI are hardly affected at all, suggesting that the influence of a shift in endo-epi ratio is only minor, and that the reduction in diastolic dominance with increasing stenosis is mainly caused by the flow rate dependence of stenosis pressure drop (3). This is further illustrated in appendix A.

The variability of most hemodynamic and cardiac parameter values in the uncertainty quantification results is slightly smaller, but reasonably close to that in the clinical study results used for comparison. Only for diastolic time fraction the variability is much smaller in the simulations than in the clinical study results. Systolic, diastolic and mean arterial pressure in the clinical study results are significantly lower than those in the simulation results, while pulse pressure and cardiac period are significantly higher. This may in part be caused by inaccuracies in estimating central pressure from peripheral pressure in the clinical study, but also the changes in hemodynamics during CABG surgery, due to anesthetics and manipulation of the heart, are expected to be of influence [24–26].

The much lower values of $Q_\text {mean}$ in the clinical study results are expected to be related to these lower pressure values, and perhaps the relatively small patient size in the (Japanese) clinical study. The combination of lower SAP, DAP, and MAP, with higher PP, may also explain the higher PI values in the clinical data. Overall, the metrics’ values show reasonable correspondence between clinical and simulation results, especially considering the fact that healthy subject parameter values were used in the simulation models. Plausible explanations for the larger variability and weaker correlation with stenosis severity of the clinical study results may be the variation in graft types and targets (single and sequential, arterial and venous grafts, to different target coronaries, possibly not all fully occluded). Additionally, measurement inaccuracy may have played a role, for example, in estimating central pressure from peripheral measurements, but also because of the sometimes large time lapse between surgery and postoperative CCT scan (median time interval from CABG surgery to CCT examination was 33 days; IQR 19–89 days; range 6–160 days).

More patient data are needed to reliably adapt the 1D/0D model to represent CABG surgery patients, and to estimate realistic ranges and distributions for the different model parameters.

## Conclusions

Among the most commonly used patency metrics, D/S-ratio (and hence, DF%) displayed the highest sensitivity to stenosis severity, and the lowest sensitivity to the remaining physiological parameters in the study. The novel DRI showed even better performance in the simulation study, but not in the clinical results, suggesting that a more accurate central pressure estimate or measurement would be needed. All diastolic dominance-based metrics appear to be relatively insensitive to autoregulation.

As pointed out by Jelenc et al. [40], all metrics’ values except $Q_\text {mean}$ depend on flow probe position. However, in our results the difference between values measured proximally and distally was only $1-2$% on average, and the corresponding Sobol indices are not significantly affected.

To further increase the practical relevance of the multiscale model used in this study, it may be extended to include different graft types and targets, autoregulation, and competitive flow. This would also allow further honing of flow-based patency metrics and their associated measurement protocol. Extending the model would require more computing power, further restriction of UQ complexity, or a more efficient model implementation. Additionally, a better understanding of hemodynamic and physiological changes during CABG surgery is needed for a more realistic model, using CABG patient parameters rather than healthy subject parameters. To this end, data from existing and new clinical studies may be used, for example, in combination with the approach adopted by Tran et al. [42].

Overall, from the angle of a company seeking to further improve and broaden the diagnostic strength of its flow-based CABG patency metrics and protocols, the method presented in this paper appears promising. Existing or newly-formulated metrics can be examined for their ability to separate the technical error conditions under control by the surgeon from the graft properties and patient conditions not under surgical control. Along with this, the statistical correlation between graft properties and patient conditions can be associated to the model variables, to define surgery/patient-specific modifiers to existing CABG patency metrics and thereby enhance their diagnostic precision. This can guide engineering development and ensuing clinical studies to validate efficacy and clinical outcomes.

## Figures and Tables

**Fig. 11:**
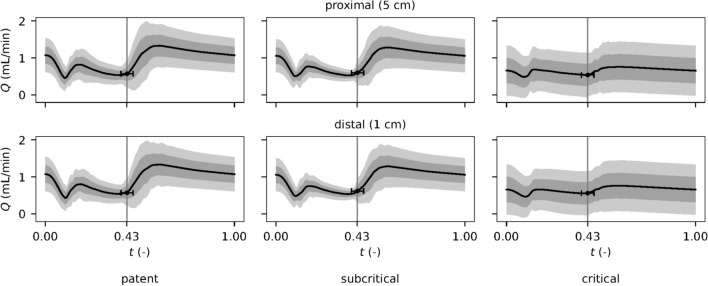
Mean LITA flow waveforms at different degrees of stenosis (for details of stenosis classes, see appendix D), determined by ensemble averaging the uncertainty quantification simulation results. Dark and light grey bands indicate one and two standard deviations, respectively. All waveforms were scaled to a duration of 1 s. The grey vertical lines indicate the start of diastole, with the errorbar indicating its standard deviation. Top row: results measured proximally (5 cm from stenosis at distal end of graft); bottom row: results measured distally (i.e. 1 cm from stenosis at distal end of graft).

**Fig. 12:**
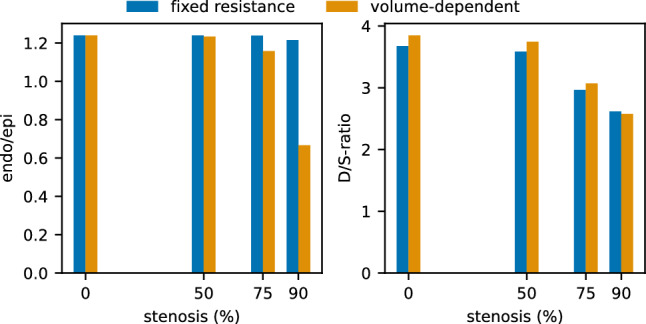
Volume-dependent intramyocardial resistance increases reduction of diastolic dominance with increasing stenosis. Top: D/S-ratio, bottom: endo/epi-ratio, both as a function of stenosis. In a model with fixed intramyocardial resistance (blue bars), endo/epi ratio is virtually independent of stenosis severity, while with volume-dependent resistance (orange bars), endo/epi ratio decreases noticeably, indicating that flow is distributed away from the subendocardium. The weaker influence of intramyocardial pressure on the subepicardium allows for more flow during systole, which results in the slightly larger reduction in D/S-ratio in the volume-dependent resistance model.

**Table 8:**
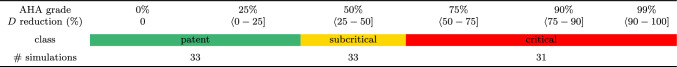
Stenosis classification criteria

**Fig. 14:**
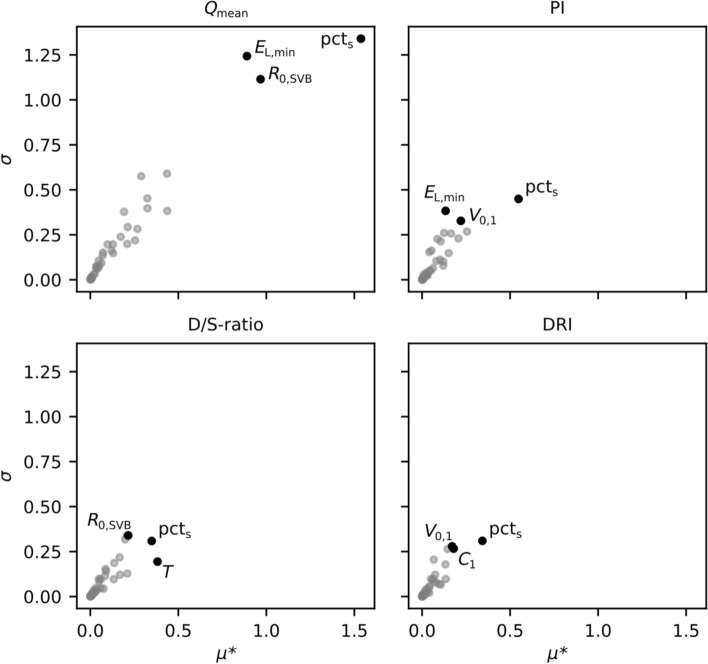
Scatter plots of Morris screening results for all metrics (results were scaled by their median values to enable comparison, most influential results are labeled).

## Data Availability

The raw results of this study are available at request from the corresponding author. For the results of the clinical study, please contact Dr. Takahashi of Nippon Medical School.

## Appendix A: Stenosis Effects

In an earlier, internal study, the 1D/0D multiscale model outlined in Sect. 2.2 was used to study the effects of stenosis. To this end, the stenosis in the LITA graft was set to 0, 50, 75 and 90%, while keeping all other parameter values at their original values, as used by Mynard et al. [16]. This allowed for detailed examination of the flow in different parts of the coronary circulation. The resulting waveforms are shown in Fig. 10. To give an indication of the variability of the waveforms at different degrees of stenosis, Fig. 11 shows the mean LITA flow waveforms with their standard deviations, determined by ensemble averaging the uncertainty quantification simulation results.

In normally functioning coronary arteries and grafts, the flow waveform is diastolic dominant, with low flow rate during systole and high flow rate during diastole. This can be explained by the contraction of the ventricles during systole, which compresses the intramyocardial vessels, squeezing out blood. This blood flows into the coronary veins, where pressure is low, while arterial inflow is inhibited. During diastole, the ventricles relax, creating a suction effect in the intramyocardial vessels, while pressure in the aorta is still relatively high (aortic valve is closed). This allows blood to flow into the coronary arteries, and replenish intramyocardial blood volume (Spaan’s intramyocardial pump model [30]).

When a graft is stenosed, its flow rate decreases with increasing stenosis severity. This decrease is stronger in diastole than in systole, leading to decreased diastolic dominance of the flow waveform (Fig. 10). In part, this can be explained by the first two terms in the equation for pressure drop in a stenosed vessel (3): the effect of stenosis is stronger during diastole, when the flow rate is high. Additionally, changes in intramyocardial perfusion play a role: stenosis causes a reduction in perfusion pressure, which leads to diameter reduction of the intramyocardial vessels, so that their resistance increases. This effect is stronger in the sub-endocardium (higher compliance [19]) than in the sub-epicardium (lower compliance), causing the flow distribution to shift towards the sub-epicardium. In the sub-epicardium, intramyocardial pressure is lower than in the sub-endocardium, and hence, systolic flow impediment is not as strong here. Therefore, the shift of the flow distribution towards the sub-epicardium results in a further reduction of diastolic dominance (Fig. 12). Finally, on the proximal side of the graft, the compliance of the graft itself influences the flow waveform, as demonstrated by Jelenc et al. [40]. The perfusion pressure gradient causes the blood volume in the graft to increase (positive gradient) or decrease (negative gradient), so that the flow rate on the proximal side exceeds that on the distal side, or vice-versa.

The patency metrics presented in Sect. 2.1 quantify different aspects of how the flow waveform changes in the presence of stenosis: $Q_\text {mean}$ quantifies the overall decrease in flow rate, while D/S-ratio, DF% and DRI capture the decrease in diastolic dominance. Finally, PI quantifies the combined effect of increasing capacitive flow proximal to the stenosis, and decreasing mean flow rate.

## Appendix B: Multi-Scale Coronary Circulation Model

The models and parameter values given in this section are based on work by Mynard et al. [14, 16]. The coronary tree and closed-loop cardiovascular model are made up of segments in which the one-dimensional (1D) Navier–Stokes equations are solved, and lumped-parameter, zero-dimensional (0D) vascular bed models.

### 1D Segments

The 1D flow equations are:

B1a $$\begin{aligned} \frac{\partial A}{\partial t} + \frac{\partial Au}{\partial t}&= 0, \end{aligned}$$

B1b $$\begin{aligned} \frac{\partial u}{\partial t} + u\frac{\partial u}{\partial x} + \frac{1}{\rho }\frac{\partial p}{\partial x}&= -\frac{\xi \pi \mu }{\rho }\frac{u}{A}, \end{aligned}$$

where *t* is time, *x* is axial position, *A* and *u* are vessel cross-section and blood flow velocity, respectively, *p* is pressure in the vessel, $\rho$ is blood density (1.06 g/cm$^3$), $\mu$ is (dynamic) blood viscosity (0.035 poise), and $\xi = 22$ is a constant for the velocity profile in the vessel. Transmural pressure, $p_\text {tm} = p - p_\text {ext}$ ($p_\text {ext}$: external pressure), is related to *A* through:

B2a $$\begin{aligned} p_\text {tm}(A)&= \frac{2\rho c_0^2}{b} \left[ \left( \frac{A}{A_0}\right) ^{b/2} - 1\right] + p_0, \end{aligned}$$

B2b $$\begin{aligned} b&= \frac{2\rho c_0^2}{p_0 - p_\text {coll}}. \end{aligned}$$

Here, $p_0$ and $p_\text {coll}$ are reference and collapse pressure (-10 mmHg), respectively, and $A_0$ and $c_0$ are reference cross-section and wave speed at $p = p_0$, respectively.

Parameter values for the coronary artery segments (Fig. 2) and grafts are given in Table 2, while those for the closed-loop circulation model (Fig. 3) are shown in Table 3. Grafts were anastomosed to LAD-2, 0.3 cm from its proximal end. The LITA graft was given linear taper from its proximal diameter (Table 2) to 0.1674 cm at its distal end. Wave speed in both grafts was computed based on diameter, following Olufsen et al. [43]. Linear taper was applied to sa (sa1 + sa2), with distal $A_0$ and $c_0$ 30% less and 43% more than proximal values, respectively. The major pulmonary arteries (pa) were given constant wave speed, and $A_0$ linearly increasing by 50%.

**Table 2:** Coronary segment properties [14] (L/RVFW: left/right ventricular free wall)

Segment	*L* (cm)	$D_0$ (cm)	$c_0$ (cm/s)	Daughter 1	Daughter 2
LM-1	1.21	0.45	749	LAD-2	Cx-3
LAD-2	0.7	0.37	837	Diag-6	LAD-7
Cx-3	1.2	0.36	852	Marg-4	Cx-5
Marg-4	2.7	0.25	1109	(LVFW)	
Cx-5	1.6	0.30	969	(LVFW)	
Diag-6	2.4	0.24	1143	(LVFW)	
LAD-7	0.8	0.33	905	Septal-8	LAD-9
Septal-8	1.7	0.24	1143	(Septum)	
LAD-9	3.7	0.27	1048	(LVFW)	
RC-1	2.8	0.41	786	AM-2	RC-3
AM-2	1.9	0.25	1109	(RVFW)	
RC-3	2.7	0.36	852	AM-4	RC-5
AM-4	1.3	0.21	1257	(RVFW)	
RC-5	5.0	0.32	925	(Septum)	
LITA	20.0	0.28	1028	LAD-2

**Table 3:** Parameter values for closed-loop circulation model in Fig. 3 (normotensive)

Segment	*L* (cm)	$A_0$ (cm$^2$)	$c_0$ (cm/s)	$p_0$ (mmHg)
ar	1	6.8	400	80
sa1	8	6.8	400	80
sa2	22	6.8	400	80
pa	5	7.1	250	11
sv1	10	6	150	5
sv2	5	6	150	5
pv	5	10	150	6

### Coronary Microvascular Bed Model

The lumped-parameter electrical circuit model displayed in Fig. 5 (main text) results in a set of ordinary differential equations (ODEs), in which the resistances $R_1$, $R_\text {m}$ and $R_2$ in each layer (subepicardium, midwall, subendocardium) depend on volume, as:

B3a $$\begin{aligned} R_i&= R_{0,i}\frac{V_{0,i}^2}{V_i^2}, \end{aligned}$$

B3b $$\begin{aligned} R_\text {m}&= R_\text {0,m}\left[ \gamma \frac{V_{0,1}^2}{V_1^2} + (1-\gamma )\frac{V_{0,2}^2}{V_2^2}\right] \end{aligned}$$

B3c $$\begin{aligned} V_i (t)&= V_{0,i} + \int _0^t C_i \frac{\textrm{d}p_{\text {tm,}i}}{\textrm{d}t'} \textrm{d}t', \end{aligned}$$

where $\gamma = 0.75$, $i = [1, 2]$ indicates quantities on the arterial and venous side of the model, respectively, and subscript 0 indicates reference value. Total resistance for each layer ($R_1 + R_\text {m} + R_2$) was tuned iteratively, to achieve the target mean flow rates and endo/epi-ratios in Table 4. Myocardial weight and flow were then distributed among the different instances of the 0D-model according to Murray’s law.

**Table 4:** Parameter values for intramyocardial bed model

	LVFW	RVFW	Septum
Target mean flow rate (% CO)	2.54	0.66	1.35
Myocardial weight (g)	104	46	54
Endo/epi flow (−)	1.24	1.18	1.37
	All regions		
$C_1$ (mL$\cdot$mmHg$^{-1}\cdot 100$g$^{-1}$)	0.013		
$C_2$ (mL$\cdot$mmHg$^{-1}\cdot 100$g$^{-1}$)	0.254		
$V_{0,1}$ (mL/100 g)	2.5		
$V_{0,2}$ (mL/100 g)	8.0		
Endo/epi $V_{0,i}$, $C_i$ (-)	1.14		

(% CO: percentage cardiac output)

Intramyocardial pressure $p_\text {im}$ is composed of cavity-induced extracellular pressure (CEP) and shortening-induced intracellular pressure (SIP):

B4 $$\begin{aligned} p_\text {im} = \text {CEP} + \text {SIP}. \end{aligned}$$

Assuming a linear decrease of CEP, from cavity pressure on the endocardium to pericardial pressure (assumed to be 0) in the epicardium, $5/6\times$, $1/2\times$, and $1/6\times$CEP was applied to the subendocardium, midwall, and subepicardium, respectively. For the ventricular septum, CEP was set to vary between $p_\text {LV}$ and $p_\text {RV}$ in a similar manner. In the left ventricular free wall and septum, SIP$= \phi E_\text {LV}$, while in the right ventricular free wall, SIP$= \phi E_\text {RV}$, where $E_\text {L/RV}$ is left/right ventricular elastance, and $\phi = 7$ mL is an empirical constant.

Penetrating arterial and venous characteristic impedance, $Z_\text {pa}$ and $Z_\text {pv}$, respectively, were computed from parameters of the connecting arterial segment, as:

B5 $$\begin{aligned}&Z_\text {pa} = \frac{\rho c_0}{A_0},&Z_\text {pv} = \tfrac{1}{2} Z_\text {pa}. \end{aligned}$$

### Systemic and Pulmonary Vascular Bed Models

Systemic and pulmonary vascular beds were modeled as 3-element windkessels, with additional compliance and characteristic impedance elements on the venous side (Fig. 3, main text). The capillary (i.e. middle) resistance in these models was taken to be pressure-dependent, as:

$$\begin{aligned} R_\text {cap} (p) = \left\{ \begin{array}{l l} R_0 \left( \frac{p_\text {tm,0} - p_\text {zf}}{p_\text {tm} - p_\text {zf}}\right) , &  p_\text {tm} > p_\text {zf}, \\ \infty &  p_\text {tm} \le p_\text {zf}, \end{array} \right. \end{aligned}$$

where $p_\text {zf}$ represents zero-flow pressure, to account for the Starling mechanism [14]. Parameter values used for the systemic and pulmonary vascular beds are given in Table 5.

**Table 5:** Parameter values used for systemic (SVB) and pulmonary (PVB) vascular beds

Bed	$R_0$	$p_\text {zf}$	$C_\text {art}$	$C_\text {ven}$
	(mmHg$\cdot$s$^{-1}$mL$^{-1}$)	(mmHg)	(mL/mmHg)	(mL/mmHg)
SVB	1.0	5	1.0	11
PVB	0.05	0	5.0	15

### Heart and Valves

For the heart chambers, the relation between cavity pressure *p*(*t*) and volume *V*(*t*) was modeled using time-varying elastance *E*(*t*) [44]:

B6 $$\begin{aligned} p = E(V - V_0)\left[ 1 - \text {K}_\text {S} Q\right] , \end{aligned}$$

where $V_0$ is the pressure axis intercept of the pressure-volume relation, and K$_\text {S}$ is a source resistance constant (units s/mL). Elastance is modeled as a double Hill function, following Stergiopulos et al. [45]:

B7 $$\begin{aligned} E(t) = k \left( \frac{g_1}{1 + g_1} \right) \left( \frac{1}{1 + g_2} \right) + E_\text {min}, \end{aligned}$$

with

$$\begin{aligned}&g_1 = \left( \frac{t - t_\text {c}}{\tau _1}\right) ^{m_1},&g_2 = \left( \frac{t - t_\text {c}}{\tau _2}\right) ^{m_2}, \end{aligned}$$

where $\tau _1$ and $\tau _2$ are time constants, and $t_\text {c}$ indicates onset time. Finally, *k* is a scaling factor, given by:

$$\begin{aligned} k = \frac{E_\text {max} - E_\text {min}}{\text {max}\left[ \left( \frac{g_1}{1 + g_1}\right) , \left( \frac{1}{1 + g_2}\right) \right] }. \end{aligned}$$

As explained in the main text, chamber interaction was not included. Parameter values for this model at reference cardiac period $T = 0.89$ s are given in Table 6. To incorporate the dependence of diastole duration on heart rate, the relation determined by Salvi et al. [21] was used. To this end, the elastance function was scaled following Suga et al. [20], by first normalizing time and elastance in the reference situation by $t/t_\text {max}$ and $E/E_\text {max}$, respectively, where $t_\text {max}$ is the time at which $E_\text {max}$ is reached (approximately equal to systole duration). Next, $t_\text {max}$ at target cardiac period was determined from $t_\text {max} = 0.132 T + 0.184$ for the ventricles, while for the atria $t_\text {max}$ was assumed to be $\nicefrac {1}{3}$ of the ventricular value. Elastance at target cardiac period was determined from the normalized curve as

$$\begin{aligned} E = E_\text {max} E_\text {N}\left( \frac{t}{t_\text {max}}\right) . \end{aligned}$$

**Table 6:** Heart chamber parameter values at reference cardiac period $T = 0.89$ s

		LV	RV	LA	RA
$E_\text {max}$	(mmHg/mL)	2.8	0.45	0.13	0.09
$E_\text {min}$	(mmHg/mL)	0.070	0.035	0.090	0.045
$V_0$	(mL)	10	40	3.0	7.0
$V_\text {i}$	(mL)	136	172	71	67
$\text {K}_\text {S}$	($10^{-3}$ s/mL)	0.5	1.0	0.25	0.5
$\tau _1$	(s)	0.215	0.215	0.042	0.042
$\tau _2$	(s)	0.362	0.362	0.138	0.138
$m_1$	(−)	1.32	1.32	1.99	1.99
$m_2$	(−)	21.9	21.9	11.2	11.2
$t_\text {c}$	(s)	0	0	0.65	0.65

LV: left ventricle, RV: right ventricle, LA: left atrium, RA: right atrium, $V_\text {i}$: initial volume

The pressure drop across the heart valves was modeled by the Bernoulli equation [44]:

B8 $$\begin{aligned} \Delta p = B Q |Q| + L \frac{\textrm{d}Q}{\textrm{d}t}, \end{aligned}$$

with *B* and *L* Bernoulli resistance and blood inertance, respectively:

$$\begin{aligned}&B = \frac{\rho }{2 A_\text {eff}^2},&L = \frac{\rho l_\text {eff}}{A_\text {eff}}. \end{aligned}$$

Here, $A_\text {eff}$ and $l_\text {eff}$ are effective valve area and length, respectively. Instantaneous $A_\text {eff} (t)$ was set to depend on valve state $\zeta \in [0, 1]$ as:

$$\begin{aligned} A_\text {eff} (t) = A_\text {max} \zeta (t), \end{aligned}$$

where $A_\text {max}$ is the effective area when the valve is fully opened. By also including a (time-dependent) minimum area, effects of valve regurgitation may be incorporated, but this was omitted in our work, just like a reduction of $A_\text {max}$ to account for valve stenosis. Valve state is governed by a pressure drop dependent ODE:

$$\begin{aligned}&\frac{\textrm{d}\zeta }{\textrm{d}t} = (1 - \zeta )\text {K}_\text {vo} \Delta p,&\Delta p > 0, \\&\frac{\textrm{d}\zeta }{\textrm{d}t} = \zeta \text {K}_\text {vc} \Delta p,&\Delta p \le 0, \end{aligned}$$

with K$_\text {vo}$ and K$_\text {vc}$ rate coefficients for valve opening and closure, respectively. Parameter values for the valves used in our work are given in Table 7.

**Table 7:** Valve parameter values

		AV	PV	MV	TV	VV
$A_\text {max}$	(cm$^2$)	6.8	7.1	7.7	8.0	6.0
$l_\text {eff}$	(cm)	1.5	1.0	0.8	0.8	1.5
K$_\text {vo}$	(cm$^2$s$^2$g$^{-1}$)	0.012	0.02	0.03	0.03	0.03
K$_\text {vc}$	(cm$^2$s$^2$g$^{-1}$)	0.012	0.02	0.04	0.04	0.03

AV: aortic valve, PV: pulmonary valve, MV: mitral valve, TV: tricuspid valve, VV: venous valve

## Appendix C: Lumped-Parameter Circulation Model

Under slowly varying conditions, the 1D system of equations (B1) can be simplified into a 0D lumped-parameter model by integrating over the axial coordinate (see e.g. Formaggia and Veneziani [46]). Also referred to as electrical analog models, such models are made up of electrical circuit resistance, inductance, and capacitance components, which represent (viscous) resistance, inertia, and vessel wall compliance. These are related to a blood vessel’s geometric and mechanical properties as:

$$\begin{aligned}&C = \frac{3\pi R_0^3 l}{2 E h},&L = \frac{\rho l}{\pi R_0^2},&\quad &R = \frac{8 \mu l}{\pi R_0^4}, \end{aligned}$$

where $R_0$ and *l* are the vessel’s equilibrium radius and length (assumed constant), *E* is the vessel wall’s elasticity modulus, *h* is the vessel wall thickness, $\rho$ and $\mu$ are the density and dynamic viscosity of blood, respectively, and a parabolic velocity profile has been assumed.^2^

For Avanzolini’s lumped-parameter closed loop cardiovascular circulation model, the same parameter values were used as in the original paper, which is available online. In the Morris screening simulations, these values were used as the means for the normal distributions from which the trajectories were sampled, as they showed reasonable agreement with values reported in literature (e.g. Charlton et al. [36]). Unfortunately, due to the model structure, it was not possible to recover the underlying vessel lengths and diameters from the *R*, *L* and *C* values used by Avanzolini. For the single instance of the intramyocardial circulation model that was connected to Avanzolini’s model, the parameter values shown in Table 4 were used as means, with a standard deviation of 25% of the mean. Similar to the multi-scale model, the intramyocardial resistances were tuned iteratively to achieve the LVFW target mean flow rate and endo/epi ratio, also shown in Table 4. Waveforms and stenosis dependence resulting from this model show satisfactory agreement in terms of order of magnitude and trends (Fig. 13). Furthermore, it should be noted that, when the ascending aorta pressure waveform is replaced by that produced by that from the 1D/0D multiscale model, resulting coronary waveforms are practically indistinguishable. The complete OpenModelica implementation may be obtained from the corresponding author, at sita.drost@transonic.com.

## Appendix D: Clinical Study

The dataset provided by Takahashi et al. [11] consisted of graft flow, radial artery pressure, and ECG waveforms, measured intraoperatively in 123 grafts, in 41 patients undergoing off-pump CABG surgery. Additionally, in all grafts percentage stenosis was measured postoperatively, using multidetector computed tomography (MDCT). This prospective observational study was approved by the review board of Nippon Medical School Institutional (No. 30–11-1029) on May 15, 2019. All patients provided a written informed consent for the publication of study data.

### D.1: Measurement Protocol

After completion of a bypass conduit, first standard graft patency assessment was performed, following Japanese guidelines [8], using a Medistim VeriQ system, and evaluating based on $Q_\text {mean}$ and PI values. Next, two additional measurements were done using a Transonic AureFlo system: one with the native artery snared, and one after the snare was removed. In case of sequential grafts, measurements were taken after completion of each anastomosis. All flow measurements were taken approximately 1 cm of the distal anastomosis. The Transonic AureFlo system was connected to the vital signs monitor to measure real-time arterial pressure measured via an arterial line placed in the radial or femoral artery.

For the computation of $Q_\text {mean}$, PI, and DF%, the measurements without snare were used, whereas for the computation of DRI the measurement with highest $Q_\text {mean}$ was used.

### D.2: Patency Classes

Based on postoperative MDCT measurements, percentage stenosis in all grafts was determined. Grafts were classified as patent, subcritically stenosed, or critically stenosed, according to the criteria outlined in Table 8.

## Appendix E: Morris Screening

For all metrics, the Morris screening results are plotted in Fig. 14. The 5 most influential parameters are listed in Table 9, with the corresponding values of $\sqrt{\mu ^{*2} + \sigma ^2}$ (before computing $\mu ^*$ and $\sigma$, results were scaled by their median values, to enable comparison between metrics). For all metrics, percentage stenosis is the most influential parameter.

**Table 9:** Morris sensitivity measures, $\sqrt{\mu ^{*2} + \sigma ^2}$ (dimensionless)

$Q_\text {mean}$		PI		D/S-ratio		DRI	
pct$_\text {s}$	2.04	pct$_\text {s}$	0.71	pct$_\text {s}$	0.47	pct$_\text {s}$	0.46
$E_\text {L,min}$	1.53	$E_\text {L,min}$	0.41	*T*	0.43	$V_{0,1}$	0.33
$R_\text {0,SVB}$	1.48	$V_{0,1}$	0.39	$R_\text {0,SVB}$	0.40	$C_1$	0.32
$E_\text {L,max}$	0.73	$R_\text {0,SVB}$	0.37	$C_1$	0.40	$R_\text {0,SVB}$	0.30
$V_{0,1}$	0.64	$C_1$	0.31	$V_{0,1}$	0.37	$w_\text {L}$	0.22

pct$_\text {s}$: percentage stenosis, $E_\text {L,min}$: min. left ventricular elastance, $E_\text {L,max}$: max. left ventricular elastance, $R_\text {0,SVB}$: reference resistance, systemic vascular bed, $V_{0,1}$: reference intramyocardial volume, arterial side, $C_1$: intramyocardial compliance, arterial side, $w_\text {L}$: myocardial weight, left

## Appendix F: Sobol Indices

Sobol indices for all metrics are displayed in Table 10 (proximal measurements) and Table 11 (distal measurements). Because for all metrics, a linear gPCE was selected, main and total Sobol indices are equal, and only one value is displayed in the tables. It should be noted that $Q_\text {mean}$, and its corresponding Sobol indices must be independent of flow probe position. The small differences between proximal and distal Sobol indices for $Q_\text {mean}$ are expected to be caused by numerical inaccuracy. Therefore, comparable numerical inaccuracy-related differences should be expected between the proximal and distal Sobol indices for the other metrics.

**Table 10:** Sobol indices, from 9-parameter gPCE (monitored 5 cm from stenosis; most influential printed in bold)

	$Q_\text {mean}$	PI	D/S-ratio	DRI
pct$_\text {s}$	$3.19\times 10^{-1}$	$5.23\times 10^{-2}$	$\mathbf {3.64\times 10^{-1}}$	$\mathbf {6.83\times 10^{-1}}$
$R_\text {0,SVB}$	$1.52\times 10^{-1}$	$2.30\times 10^{-2}$	$3.01\times 10^{-2}$	$2.78\times 10^{-3}$
$A_\text {0,sa}$	$3.11\times 10^{-4}$	$2.78\times 10^{-2}$	$7.45\times 10^{-2}$	$5.17\times 10^{-3}$
$c_\text {0,sa}$	$2.78\times 10^{-3}$	$3.12\times 10^{-2}$	$1.47\times 10^{-1}$	$9.07\times 10^{-4}$
$A_\text {0,lita}$	$\mathbf {4.60\times 10^{-1}}$	$1.30\times 10^{-1}$	$1.32\times 10^{-1}$	$1.91\times 10^{-1}$
$c_\text {0,lita}$	$4.22\times 10^{-4}$	$9.96\times 10^{-2}$	$5.48\times 10^{-4}$	$1.61\times 10^{-3}$
$C_1$	$5.96\times 10^{-4}$	$2.39\times 10^{-3}$	$1.22\times 10^{-2}$	$3.26\times 10^{-2}$
$V_{0,1}$	$8.03\times 10^{-4}$	$1.41\times 10^{-1}$	$4.11\times 10^{-3}$	$5.21\times 10^{-2}$
*T*	$6.36\times 10^{-2}$	$\mathbf {4.92\times 10^{-1}}$	$2.36\times 10^{-1}$	$3.06\times 10^{-2}$

pct$_\text {s}$: Percentage stenosis; $R_\text {0,SVB}$: reference resistance systemic vascular bed; $A_\text {0,sa}$: unstressed cross section systemic artery; $c_\text {0,sa}$: unstressed wave speed systemic artery; $A_\text {0,lita}$: unstressed cross section LITA graft; $c_\text {0,lita}$: unstressed wave speed LITA graft; $C_1$: intramyocardial compliance (arterial side); $V_{0,1}$: reference intramyocardial volume (arterial side); *T*: cardiac period

**Table 11:** Sobol indices, from 9-parameter gPCE (monitored 1 cm from stenosis; most influential printed in bold)

	$Q_\text {mean}$	PI	D/S-ratio	DRI
pct$_\text {s}$	$3.19\times 10^{-1}$	$1.40\times 10^{-1}$	$\mathbf {3.64\times 10^{-1}}$	$\mathbf {6.91\times 10^{-1}}$
$R_\text {0,SVB}$	$1.52\times 10^{-1}$	$2.03\times 10^{-2}$	$2.84\times 10^{-2}$	$2.40\times 10^{-3}$
$A_\text {0,sa}$	$3.12\times 10^{-4}$	$2.72\times 10^{-2}$	$7.17\times 10^{-2}$	$4.33\times 10^{-3}$
$c_\text {0,sa}$	$2.77\times 10^{-3}$	$1.97\times 10^{-2}$	$1.45\times 10^{-1}$	$2.00\times 10^{-3}$
$A_\text {0,lita}$	$\mathbf {4.60\times 10^{-1}}$	$1.28\times 10^{-1}$	$1.32\times 10^{-1}$	$2.01\times 10^{-1}$
$c_\text {0,lita}$	$4.20\times 10^{-4}$	$9.78\times 10^{-2}$	$3.60\times 10^{-3}$	$2.31\times 10^{-5}$
$C_1$	$5.97\times 10^{-4}$	$5.65\times 10^{-3}$	$1.21\times 10^{-2}$	$3.11\times 10^{-2}$
$V_{0,1}$	$8.02\times 10^{-4}$	$1.19\times 10^{-1}$	$4.29\times 10^{-3}$	$4.64\times 10^{-2}$
*T*	$6.36\times 10^{-2}$	$\mathbf {4.43\times 10^{-1}}$	$2.39\times 10^{-1}$	$2.16\times 10^{-2}$

pct$_\text {s}$: Percentage stenosis; $R_\text {0,SVB}$: reference resistance systemic vascular bed; $A_\text {0,sa}$: unstressed cross section systemic artery; $c_\text {0,sa}$: unstressed wave speed systemic artery; $A_\text {0,lita}$: unstressed cross section LITA graft; $c_\text {0,lita}$: unstressed wave speed LITA graft; $C_1$: intramyocardial compliance (arterial side); $V_{0,1}$: reference intramyocardial volume (arterial side); *T*: cardiac period

## Footnotes

1 Repeating the analysis with uniform distribution for all variables was not feasible because of restricted time, but would be interesting for a future study.

## Code Availability

The Python and Modelica code used forstudy are available at request from the corresponding author.
